# Lyme Disease Incidence in Massachusetts, 2012-2024

**DOI:** 10.1001/jamanetworkopen.2025.47392

**Published:** 2025-12-05

**Authors:** Selsebil Sljivo, Michael Klompas, Karen Eberhardt, Tom Chen, Liisa M. Randall, Catherine M. Brown, Noelle M. Cocoros

**Affiliations:** 1 Department of Population Medicine, Harvard Pilgrim Health Care Institute, Boston, Massachusetts; 2 Department of Medicine, Brigham and Women’s Hospital, Boston, Massachusetts; 3 Harvard Medical School, Boston, Massachusetts; 4 Commonwealth Informatics, Waltham, Massachusetts; 5 Department of Biostatistics, Harvard T.H. Chan School of Public Health, Boston, Massachusetts; 6 Massachusetts Department of Public Health, Boston

## Abstract

This cross-sectional study examines trends in Lyme disease incidence using data from the Massachusetts Department of Public Health's electronic health record–based surveillance platform.

## Introduction

Lyme disease is the most common vector-borne disease in the US,^1^ endemic in Massachusetts,^2,3^ and potentially increasing.^4,5^ We used electronic health record (EHR) data obtained via the Massachusetts Department of Public Health’s automated EHR-based surveillance platform, Electronic medical record Support for Public health (ESP), to investigate Lyme disease trends.

## Methods

We evaluated incident Lyme disease from January 2012 through December 2024 among 5 clinical practice groups (grouped alphabetically as A through E) in eastern Massachusetts that collectively care for approximately 30% of the state population. Lyme disease was defined using a validated algorithm^2^: (1) Lyme diagnosis code and prescription within 14 days for 7 or more days of antibiotics; (2) positive Lyme Western Blot; (3) positive Lyme polymerase chain reaction test; or (4) positive modified 2-tiered test. We calculated Lyme incidence overall, by practice, and algorithm component and evaluated trends. See Supplement 1 for full Lyme criteria and statistical methods. Periods were classified as stable when equivalence-test *P* < .05; if equivalence was not established, periods were classified as increasing or decreasing when the 2-sided trend-test *P* < .05. The Harvard Pilgrim Health Care Institute institutional review board deemed this analysis public health surveillance and exempt from informed consent. This study followed the Strengthening the Reporting of Observational Studies in Epidemiology (STROBE) reporting guideline for cross-sectional studies.

## Results

**Figure 1.**
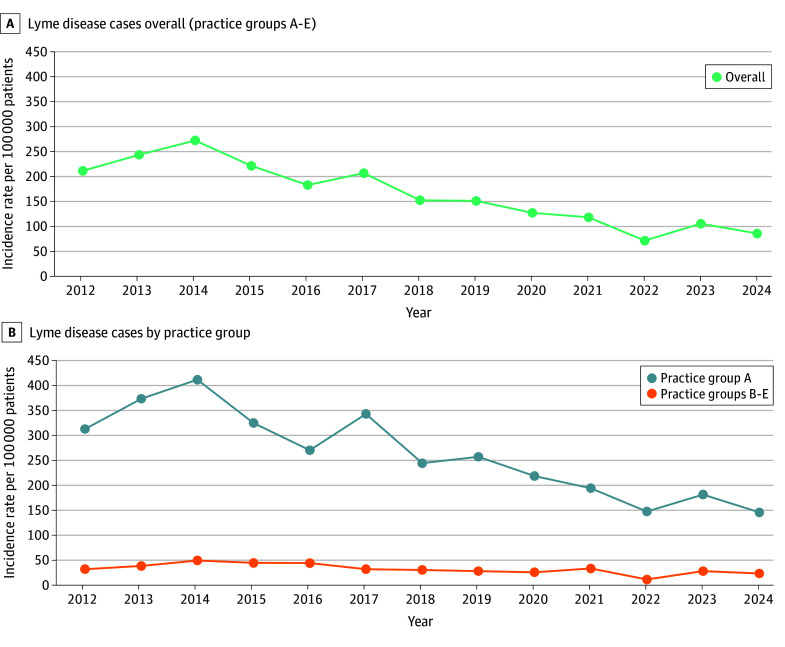
Lyme Disease Incidence Overall and by Practice Group, Massachusetts, 2012-2024 Data from 5 practice groups in eastern Massachusetts contributed to the analysis. Practice group A is the largest, serving patients in urban and suburban areas. Practice groups B through E are in urban centers.

Across all practices, there were between 1003 and 2731 Lyme cases reported per year. Lyme disease incidence declined from 211 cases per 100 000 patients in 2012 to 86 per 100 000 patients in 2024 (*P *for significance < .001) (Figure 1). However, stratifying by practice group revealed distinctions. In practice group A, the largest group, incidence rose from 314 cases per 100 000 patients in 2012 to 412 per 100 000 patients in 2014 (*P* for significance < .001), then decreased to 147 per 100 000 patients in 2024 (*P* for significance < .001). In the other 4 practice groups combined, rates were substantially lower and similar across time (33 cases per 100 000 patients in 2012, 26 per 100 000 patients in 2024) (*P* for equivalence = .006).

**Figure 2.**
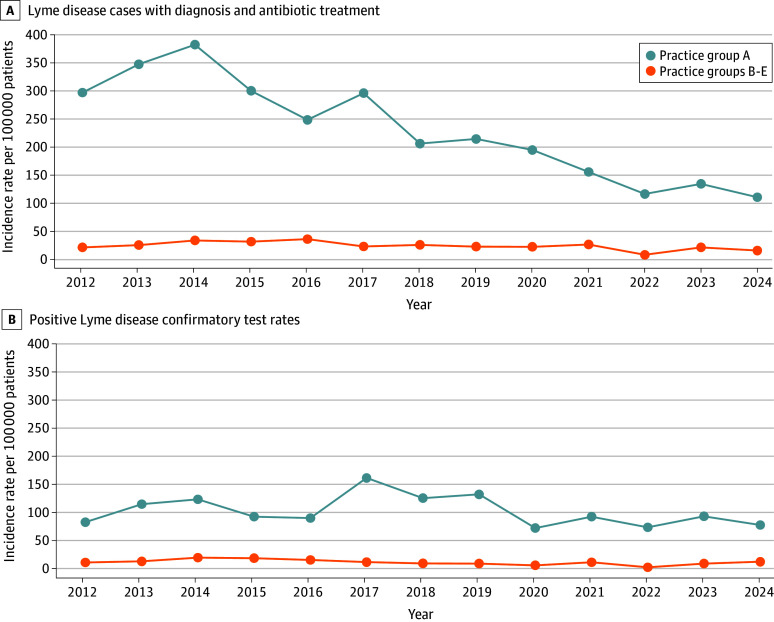
Incidence of Lyme Disease Algorithm Components by Practice Group in Massachusetts, 2012-2024 Data from 5 practice groups in eastern Massachusetts contributed to the analysis. Practice group A is the largest, serving patients in urban and suburban areas. Practice groups B-E are in urban centers.

To understand these differences between groups, we evaluated trends by algorithm component. Cases established via a diagnosis code and antibiotic prescription decreased from 2014 to 2024 in practice group A (*P* for significance < .001) but the incidence of cases with positive confirmatory tests 2014 to 2024 was stable (*P* for equivalence < .001) (Figure 2). In practice groups B through E, both these measures were stable between 2014 and 2024, both per practice and combined (diagnosis plus antibiotic: *P* for equivalence = .003; confirmatory test: *P* for equivalence = .02). On investigation, the decline in cases with a diagnosis code and antibiotic in practice group A coincided with a change in how Lyme enzyme–linked immunosorbent assay (ELISA) results were reported to practice A clinicians starting in 2015: instead of reporting screening tests as “positive” they were reported as “WesternToFollow” to discourage clinicians from prescribing antibiotics on the basis of positive screening ELISAs alone but rather to wait for confirmatory Western Blots.^6^ Subsequently, the percentage of patients in practice group A treated for Lyme without a positive confirmatory test decreased from 72% in 2012 to 40% in 2024.

## Discussion

An automated EHR-based surveillance platform including data on more than 1.2 million patients from 5 Massachusetts practice groups initially suggested a substantial decrease in Lyme incidence from 2014 to 2024. However, on further analysis the perceived decrease was likely driven by 1 large practice group that implemented a quality improvement initiative to reduce Lyme diagnoses and prescriptions for patients with positive screening tests but negative or pending confirmatory tests.^6^ The initiative’s success may explain the misleading impression that Lyme disease incidence was decreasing statewide, a trend that was not apparent when we examined trends in patients with positive confirmatory tests alone. Our findings demonstrate how practice-specific changes can impact perceived population-level disease trends, and the need for granular source data and communication with practices to elucidate observed macrotrends. Limitations of our study include the focus on a subset of practices in eastern Massachusetts and lack of medical record reviews or clinician interviews to confirm the purported mechanism underlying observed changes. While automated EHR-based public health surveillance platforms can provide rich data on disease incidence, trends, and characteristics, public health authorities must be vigilant to local initiatives and changes in clinical practice patterns that may influence perceived trends. This is true of all surveillance systems, but access to granular EHR data may facilitate explaining observed trends.

## Notes

Supplement 1. eMethods.

Supplement 2. Data Sharing Statement
