# Supplementary material for: Lyme Disease Incidence in Massachusetts, 2012-2024
Source: JAMA Netw Open. 2025 Dec 5;8(12):e2547392. doi: 10.1001/jamanetworkopen.2025.47392 (PMC12681032; doi:10.1001/jamanetworkopen.2025.47392)
Supplement: Supplement 1. — eMethods. [file jamanetwopen-e2547392-s001.pdf]

Supplemental Online Content

Sljivo S, Klompas M, Eberhardt K, et al. Artifactual changes in Lyme disease incidence in Massachusetts, 2012-2024. *JAMA Netw Open.* 2025;8(12):e2547392. doi:10.1001/jamanetworkopen.2025.47392

eMethods.

This supplemental material has been provided by the authors to give readers additional information about their work.

© 2025 Sljivo S et al. *JAMA Network Open*.

eMethods.

Data sources

The five participating groups represent a mix of ambulatory care organizations (practice group A), community health systems with ambulatory and hospital care (practice groups B and C), and federally qualified health centers (practice groups D and E) serving communities across eastern Massachusetts. Practice group A provides care to ~700,000 patients annually; the other practice groups serve ~35,000-220,000 patients annually. Practice group A serves a patient population that is both urban and suburban. The other practice groups are in urban centers.

Lyme disease surveillance algorithm

Lyme disease was defined using the following validated algorithm[1]: 1) Lyme ICD-9 (0.88.81) or ICD-10 diagnosis code (A69.20, A69.21, A69.22, A69.23, A69.29) and prescription within 14 days for ≥7 days of an indicated antibiotic (doxycycline, amoxicillin, ceftriaxone, cefotaxime, azithromycin, tetracycline, cefuroxime); 2) positive Lyme Western Blot; 3) positive Lyme PCR; or 4) positive modified two-tiered test.

Analytic methods

Annual incidence was calculated separately for each practice. Denominators were the number of unique patients with ≥1 encounter during each calendar year.

To evaluate temporal trends, we fit Poisson regression models with a log link and an offset for encounters to estimate per-year rate ratios (RRs). We applied two one-sided tests (TOST) against a prespecified region of practical equivalence for the per-year rate ratio (0.90–1.10). Periods were classified as stable when the equivalence-test p-value ($\mathrm{p_{equiv}}$) was <0.05; if equivalence was not established, periods were classified as increasing or decreasing when the two-sided trend-test p-value ($\mathrm{p_{sig}}$) was <0.05. Equivalence testing followed the TOST framework

© 2025 Sljivo S et al. *JAMA Network Open*.

and was implemented in R version 4.5.0, with all other analyses conducted in SAS version 9.4 (SAS Institute Inc).

This study is reported in accordance with STROBE reporting guidelines.

Reference

1. Nagavedu K, Eberhardt K, Willis S, Morrison M, Ochoa A, Soliva S, Scotland S, Cocoros NM, Callahan M, Randall LM, Brown CM, Klompas M. Electronic health record data for Lyme disease surveillance, Massachusetts, USA, 2017–2018. Emerg Infect Dis. 2024;30(7):1374–1379. doi:10.3201/eid3007.230942. PMID: 38916563; PMCID: PMC11210632.

© 2025 Sljivo S et al. *JAMA Network Open*.
