# Supplementary material for: Lyme Disease Incidence in Massachusetts, 2012-2024
Source: JAMA Netw Open. 2025 Dec 5;8(12):e2547392. doi: 10.1001/jamanetworkopen.2025.47392 (PMC12681032; doi:10.1001/jamanetworkopen.2025.47392)
Supplement: Supplement 2. — Data Sharing Statement [file jamanetwopen-e2547392-s002.pdf]

Data Sharing Statement

Sljivo. Artifactual Changes in Lyme Disease Incidence in Massachusetts, 2012-2024. *JAMA Netw Open*. Published December 05, 2025. doi:10.1001/jamanetworkopen.2025.47392

Data
Data available: No

Additional Information
Explanation for why data not available: Due to data privacy restrictions individual level data cannot be shared.
